# Dental Education Challenges during the COVID-19 Pandemic Period in Italy: Undergraduate Student Feedback, Future Perspectives, and the Needs of Teaching Strategies for Professional Development

**DOI:** 10.3390/healthcare9040454

**Published:** 2021-04-12

**Authors:** Giuseppe Varvara, Sara Bernardi, Serena Bianchi, Bruna Sinjari, Maurizio Piattelli

**Affiliations:** 1 Department of Innovative Technologies in Medicine and Dentistry, University of Chieti–Pescara ‘Gabriele d’Annunzio’, via dei Vestini 11, 66100 Chieti, Italy; gvarvara@unich.it (G.V.); b.sinjari@unich.it (B.S.); maurizio.piattelli@unich.it (M.P.); 2 Department of Life, Health and Environmental Sciences, University of L’Aquila, via Vetoio Coppito 2, 67100 L’Aquila, Italy; serena.bianchi@univaq.it; 3 Center of Microscopy, University of L’Aquila, via Vetoio Coppito 1, 67100 L’Aquila, Italy

**Keywords:** dentistry, education, COVID-19, public health, pandemic

## Abstract

The COVID-19 pandemic literally stopped most human movement and activities as it initially spread, which included dental practices and dental education. This defined the need for significative changes in teaching and learning with the use of “e-learning” methods, also for traineeships. This study was designed to determine the undergraduate student perception of these new methods as part of their education. This involved 353 students attending the Dental School of the G. D’Annunzio University of Chieti–Pescara, from the first to the sixth years. A questionnaire in Italian and was set-up using “Google Forms” and sent by email to the students. The questionnaire was divided into three parts: the first part included questions for general information, including age, sex and year of course; the second part had multiple choice questions related to their evaluation of the e-learning teaching, using a scale of opinion in the replies to each question (e.g., “scarce”, “fair”, “satisfying”, “very good” and “excellent”); and the third part included two open questions to indicate the strengths and limitations of these new teaching and learning approaches. The categorical variables in the first and second parts of the questionnaire were evaluated using Chi squared tests, setting significance at *p* < 0.05, while the comments were evaluated qualitatively. The student feedback showed significant appreciation (*p* < 0.05) of the new methods and the efforts that the lecturers put in to provide lectures of as high a quality as possible. However, a lack of practical training was significantly perceived as an important problem in the structure of their new curriculum (*p* < 0.05). COVID-19 has been an epic tragedy that has hit the human population not only in terms of health and healthcare, but also quality of life. This includes the quality of dental education within universities. However, the pandemic can be seen to also represent motivation to invest in the necessary technological innovation to deliver the best possible education to our future dentists.

## 1. Introduction

The appearance and spread of a new infectious disease with an abnormal behavior characterized the end of 2019 and the beginning of 2020 throughout the world. COVID-19 spread so extensively as to constitute a pandemic, as declared by the World Health Organization on 11 March 2020. This developed into a real and important public health emergency in many countries [1].

The transmission of COVID-19 is mainly respiratory, through droplets of saliva passed from human to human [2]. Therefore, after the initial period of uncertainty due to the novelty of the virus itself, the recommendations to flatten the curve of new COVID-19 infections was physical isolation and the so-called “lockdown” of all but essential social and working activities [3]. Universities were thus interrupted their teaching and practical activities in March 2020 and began to rely on the “virtual platforms” of e-learning [3].

The media and the United Nations Educational, Scientific and Cultural Organization (UNESCO) questioned with high levels of concerns what the eventual impact of these changes to university teaching would be on student education [4]. Generally, there is agreement that interruption of teaching activities will negatively affect the quality of learning for students and will consequently have impact upon their future [4]. Dental Schools were particularly affected by the COVID-19 pandemic, due to the high risk of exposure of dental operators, including dental students doing their training practice. Therefore, lectures and practical training were suspended and e-learning activities were worked on as an aid to their continued educational activities [5].

As it will be possible to return to the previous teaching patterns when the pandemic is more under control through the discovery of a valid treatment or vaccine, a period of co-existence with the virus has rapidly become the new normality. Therefore, the period where e-learning is essential for the continuation of at least the theoretical lectures and student education will need to be carefully analyzed, with a focus on the strengths and limitations and with a view to understanding what has indeed worked and what has not. This new knowledge then needs to be integrated into future improvements to the system.

The aim of this study was to evaluate the feedback from dental students on their online lectures during the initial March/April/May 2020 lockdown period and to determine their perception of their own preparation using this teaching method.

## 2. Materials and Methods

### 2.1. The New Teaching Methods

The lessons of the Dental School of the “G. D”Annunzio” University of Chieti–Pescara during the initial lockdown period through the COVID-19 emergency (from mid-March 2020) were delivered in an e-learning mode. Students and teachers used the “Teams” software (Microsoft). The University had already activated an agreement to offer the entire Office 365 package (Microsoft) for free to students and university employees. Through Teams, virtual rooms were created and made available to students and lecturers for each course. In each virtual room (generically called “Canale” or “Team”), the meetings through which lessons were held were planned. Teams made it possible to share the computer screen and the various computer files (e.g., documents, PDFs, PowerPoint files) with meeting participants, exactly as if this were a video projection in a lecture hall.

The University server organized all of the virtual rooms through the site: https://zeus.unich.it/teledidattica/ (latest access 11 April 2021) through which it was possible to trace the virtual rooms dedicated to each specific university course. Video tutorials and “Frequently Asked Questions” were made available through the same website. The server administrators could be quickly and easily contacted for clarification and technical questions using an email address and also by phone and through the Teams site itself. It was thus possible for lectures to continue through this e-learning mode, with the meetings-events planned on the Teams channels, which functioned as lecture halls (i.e., virtual rooms).

The university courses that included internships (practical training) were also carried out in e-learning mode. Indeed, Italian Law N° 22 of 8 April 2020 allows students to carry out their internship activities in different ways than before, including through this e-learning mode. The modalities for these e-learning internships were defined by the Council of Italian Dental Schools, as follows: “The internship foresees 25 h of student training for each University Training Credit (defined as CFU), of which most of these hours are individual study”. Therefore, it was possible for to the students to gain each internship CFU through specific topics on which they presented a “paper” as a PowerPoint presentation.

The topics that were assigned for this presentation and discussion in e-learning mode were those of the program of each course or of clinical cases (using, e.g., X-rays, photographs). For organizational reasons, each student was assigned 10 min for their presentation and discussion, to include any questions or clarifications from the lecturer. With reference to these methods identified by the Degree Program Council, the lecturers carried out the internships in e-learning mode by designing exercises and providing seminars that could be carried out in such a way as to allow interactions with the lecturers that were as comparable as possible to those during internships where the students were physically present at the University Dental Clinic.

Lectures and internships were carried out following the guidelines of the National Agency for the Evaluation of the University System and Research, which were issued according to Ministerial Decree 987/2016, to guarantee the highest levels of student concentration. For this, the Sector for the Coordination of Activities related to Teaching, Research and the Third Mission of the University recommended to value the opportunity of the lessons delivered in this e-learning modality and to contemplate the development of a time pause that corresponded to that observed for traditional face-to-face sessions. The degree of concentration during these lectures “at a distance”—especially when the dynamics of interactivity with the attending students were reduced—and the possibility of listening to lectures again, might have suggested shorter durations of these operations. This aspect was left to be assessed by the individual lecturers, to exercise their prerogative according to the sphere of their own didactic autonomy.

Thus, to improve student preparation, lecturers have recorded their lessons, to provide for the frequent need of the students to listen to them again. These recordings of lectures have enhanced the teaching method. The students have had the opportunity to listen to the recorded lessons through the website https://web.microsoftstream.com/ (latest access 11 April 2021).

### 2.2. Sample and Questionnaire

The sample included the 353 students who were attending the Dental School of the “G. D”Annunzio” University of Chieti–Pescara, from the first to the sixth years. The questionnaire was in Italian and it was set-up using “Google Forms” and sent by email to the students. The questionnaire was divided into three parts: the first part included questions for general information, including age, sex and year of course; the second part had multiple choice questions related to their evaluation of the e-learning teaching, using a scale of opinion for the reply to each question (e.g., “scarce”, “fair”, “satisfying”, “very good” and “excellent”); and the third part included two open questions to indicate the strengths and limitations of these new teaching and learning approaches (Table 1).

### 2.3. Ethical Considerations

The students who participated provided their informed consent in accordance with the EU General Data Protection Regulations (UE) N° 2016/679 before beginning the survey. Data collection took place from 22 May to 29 May 2020. The survey was submitted in an anonymous way through the institutional student e-mail addresses. Thus, as it was and remains, anonymous, it was not necessary to have ethical approval. The questionnaire did not contain any personal sensitive data and to complete it, the students had to give their consent to the analysis of their data.

### 2.4. Statistical Analysis

The statistical analysis included the use of descriptive statistic, for evaluation of the mean regarding age and the evaluation of the frequencies regarding the other data (e.g., gender, year of course and the multiple choice questions). The normality distribution of the given answer was assessed used Kolmogorov–Smirnov test, setting the null-hypothesis the distribution does not differ from the normal distribution [6]. After verifying the non-normality distribution (*p* < 0.05) and due to the nature of the considered variables (categorical data), for each year of the course, the answers to the multiple choice questions were evaluated using Pearson”s chi-squared tests, considering as null-hypothesis there would be no differences in the given answer, setting significance at *p* < 0.05. Finally, the data from the multiple choice questions from all the considered years were evaluated using Kruskal–Wallis tests, considering as null-hypothesis there would be no differences in the given answers, setting as dependent variable as the year of the course and the significance at *p* < 0.05. The statistical analyses procedures included the use of the SAS OnDemand for Academics software (SAS Institute, Cary, NC, USA).

In addition, a stakeholder mapping matrix has been performed to focus on those key-characters who would be involved to manage and solve the issues raised from the new model of dental education.

The comments from the third part of the questionnaire were evaluated qualitatively; more than two similar statements were grouped, to better understand the needs and the further steps to improve the e-teaching and e-learning quality.

## 3. Results

### 3.1. Demographic Characteristics and Results from the Different Years of Course

**Figure 1:**
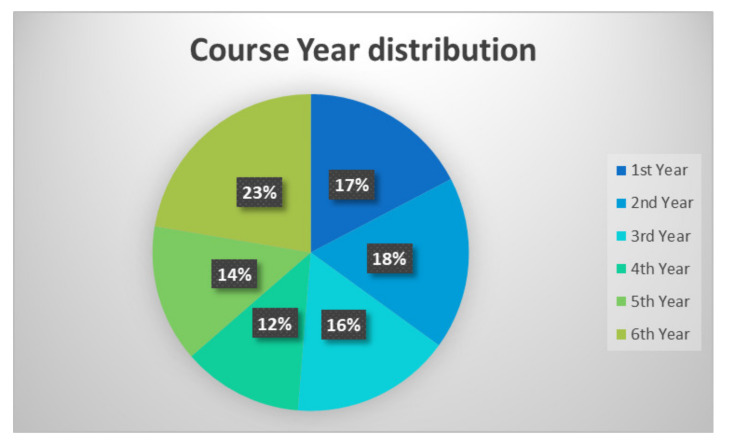
Years of the course of the responding students (*n* = 301).

From the 353 students who were invited to take part in the questionnaire, 301 accepted and completed it. Of these, 47% were female and 53% were male, with mean age of 24.2 years (range, 19–45 years). As shown in Figure 1, the proportions (%) for the distribution of the students according to their year of course were relatively similar, with the majority in their 6th year and the minority in their 4th year.

**Figure 2:**
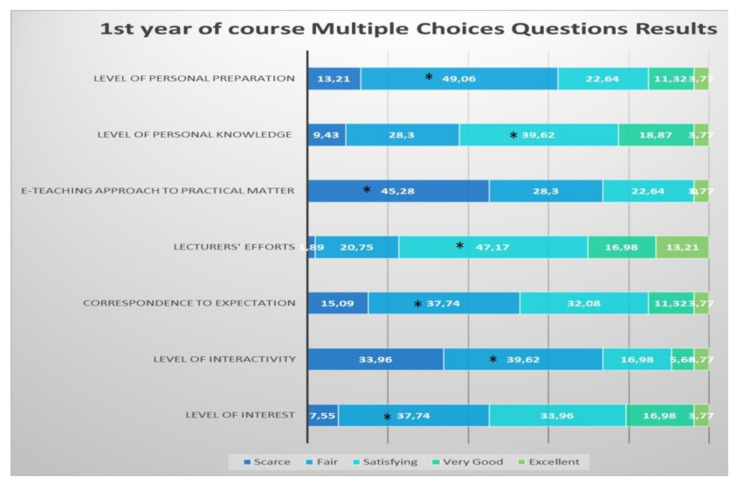
Questionnaire results for the first year student feedback to the e-learning and e-teaching approach. The numbers in the bars indicate the percentages of the answers received (*n* = 301). * *p* < 0.05 (Pearson’s chi-squared tests).

Student from the first year defined as significantly “fair” the level of interest, level of interactivity, correspondence to expectations and level of personal preparation; they defined as significantly “satisfying” the lecturers” efforts and the level of personal knowledge. These students defined as significantly “scarce” the e-teaching approach to the practical aspects of the course (Figure 2).

**Figure 3:**
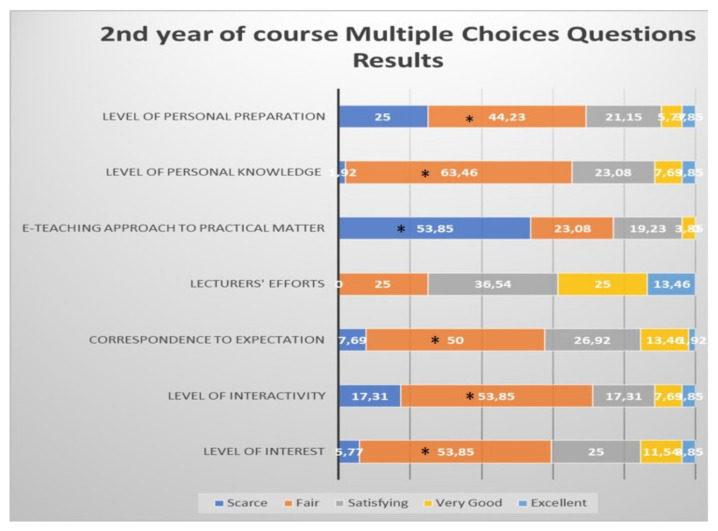
Questionnaire results for the second year student feedback to the e-learning and e-teaching approach. The numbers in the bars indicate the percentages of the answers received (*n* = 301), * *p* < 0.05 (Pearson’s chi-squared tests). Although they defined the lecturers’ efforts mostly “satisfying”, the difference did not reach statistical significance.

Students from the second year defined as significantly “fair” the level of interest, level of interactivity, correspondence to expectations and level of personal knowledge and preparation; they defined as “scarce” the e-teaching approach to the practical aspects of the course (Figure 3).

**Figure 4:**
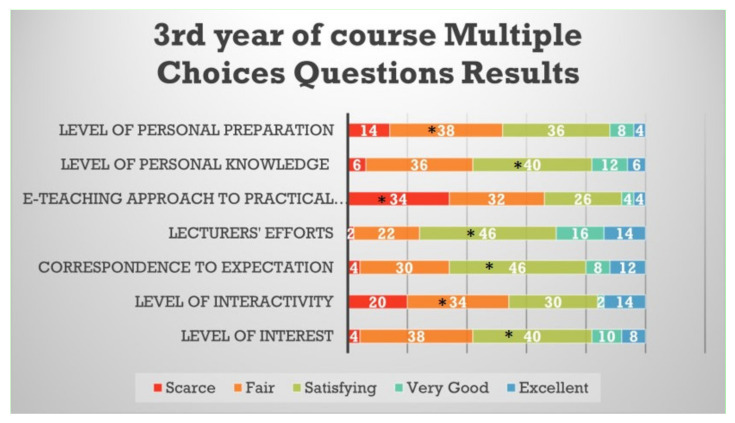
Questionnaire results for the third year student feedback to the e-learning and e-teaching approach. The numbers in the bars indicate the percentages of the answers received (*n* = 301). * *p* < 0.05 (Pearson’s chi-squared tests).

Students from the third year defined as significantly “fair” the level of interactivity and level of personal preparation and as significantly “satisfying” for the level of interest, correspondence to expectations, lecturers’ efforts and level of personal knowledge. These students from the third year defined as significantly “scarce” the e-teaching approach to the practical aspects of the course (Figure 4).

**Figure 5:**
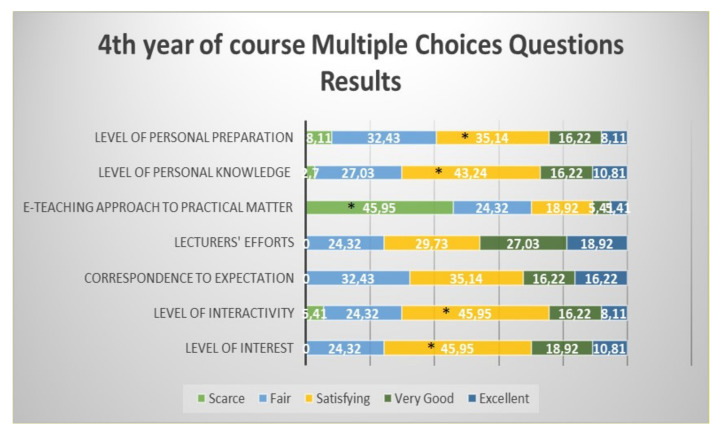
Questionnaire results for the fourth year student feedback to the e-learning and e-teaching approach. The numbers in the bars indicate the percentages of the answers received (*n* = 301). * *p* < 0.05 (Pearson’s chi-squared tests). Although the correspondence to expectations and lecturers” efforts were mostly “satisfying”, the difference did not reach statistical significance.

Students from the fourth year defined as significantly “satisfying” the level of interest, level of interactivity, level of knowledge and level of personal preparation; the e-teaching approach to practical aspects of the course was defined as significantly “scarce” (Figure 5).

**Figure 6:**
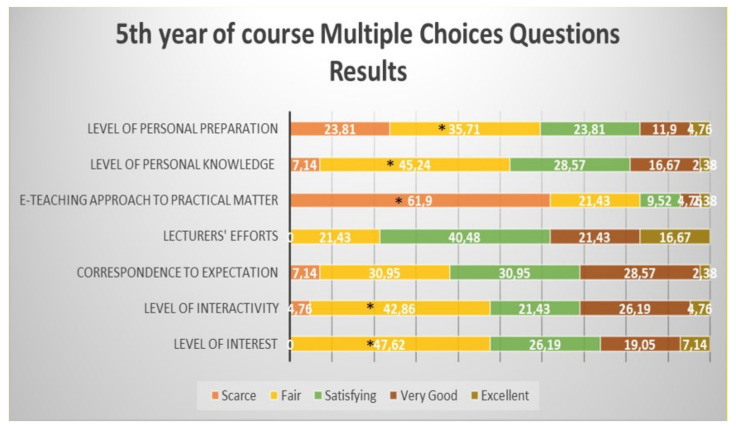
Questionnaire results for the fifth-year student feedback to the e-learning and e-teaching approach. The numbers in the bars indicate the percentages of the answers received (*n* = 301). * *p* < 0.05 (Pearson’s chi-squared tests). Although the correspondence to expectations and the lecturers” efforts were mostly defined as “satisfying”, the differences did not reach statistical significance.

Students from the fifth year defined as significantly “fair” the level of interest, level of interactivity, level of knowledge and level of personal preparation; the e-teaching approach to practical aspects of the course was defined as significantly “scarce” (Figure 6).

**Figure 7:**
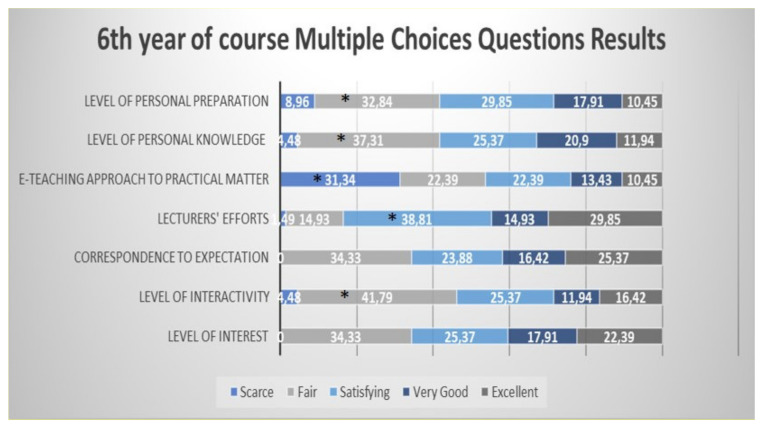
Questionnaire results for the sixth year student feedback to the e-learning and e-teaching approach. The numbers in the bars indicate the percentages of the answers received (*n* = 301). * *p* < 0.05 (Pearson’s chi-squared tests). Although the level of interest and correspondence to expectations were mostly defined as “fair”, the difference did not reach statistical significance.

Students from the sixth year defined as significantly “fair” the level of interactivity, level of knowledge and level of personal preparation; the e-teaching approach to practical aspects of the course was defined as significantly “scarce” and the lecturers’ efforts were defined as significantly “satisfying” (Figure 7).

### 3.2. The Combined Results

**Figure 8:**
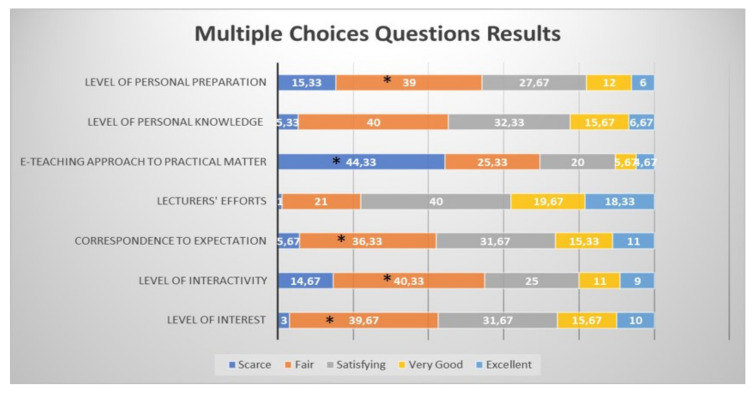
Questionnaire results for the overall combined student feedback to the e-learning and e-teaching approach, from the first to the sixth year of the course, inclusive. The numbers in the bars indicate the percentages of the answers received (*n* = 301). * *p* < 0.05 (Kruskal–Wallis tests).

The Kruskal–Wallis tests showed that the students considered significantly “fair” the level of interest, level of interactivity, correspondence with their own expectations and level of personal preparation. In addition, according to the Kruskal–Wallis tests (Table 2), the e-teaching approach to practical aspects of the course was considered significantly “scarce”. Although the perception of their own level of acquired knowledge was judged mainly “fair” by the students overall and the level of effort of the lecturers was mostly “satisfying” (Figure 8), these did not reach statistical significance.

### 3.3. The Stakeholder Mapping Matrix

The stakeholder mapping matrix highlighted how the principal characters who can contribute to improve and benefit from the improvement of the limitations of the dental educations during pandemic crisis are the institutions board components, the academic staff, the students, the providers of the online platforms and of the educational tools available to continue the training from home.

Students should be stimulated with a diversified teaching approach from the lecturers, the administrations should negotiate with the educational tool providers to assure to each students the appropriate materials to practice at home and a strict medical surveillance with a strict scheduling should be applied to assure a safe in presence traineeship (Figure S1).

### 3.4. The Open-Ended Questions: The Voices of the Students

The open-ended questions highlighted the strengths and the limitations of this new approach. Among the strength points, the more cited by the students were the feasibility of “attending” the lectures, the higher quality of the presentations due to few problems than with the lecture halls and their lighting, the possibility to record the lectures and, therefore, to listen to them again, to better understand a difficult topic and the better management of their own time to better schedule their days and their study sessions.

Among the limitations, the students raised the problem of low-quality internet connections, the lack of social interactions, the difficulties for interactivity due to little possibility for discussions with the lecturers or their peers and the lack of the practical training and internships (Table 3).

## 4. Discussion

### 4.1. The Main Issue from the Survey: How Did We Fill the Gap?

Filling the gap created by the lack of the traineeships has been challenging for the lecturers and professors. Beyond the need to re-invent without any full preparation, also to overcome the obstacles caused by distance, legal and ethical problems (regarding the patient clinical pictures rise due to the “show and share” digital environment) represented something new to be faced in a very particular situation.

On the other hand, the feedback on the attempts made to fill the gap in the practical training was negative across all the years of the course, indicating the recognized need for clinical practice for a complete dental curriculum from the first year, including the skills training and the patient interactions.

A similar survey conducted in a German dental school and recently published confirmed the trend emerged from our data. Indeed, if the online lectures were appreciated for owned qualities such as time feasibility, occasion to re-follow the lectures to better understand difficult complexes and the easy access to the shared materials, the lack of “face-to-face” training days, the “hands-on” clinical situation represents fundamental moments in the formation pathway of the dental curricula [7]. The German students perceived their practical preparation not enough to make them feel comfortable to face the future profession [7].

Another study conducted in India, not only highlighted how most of the students wished a return to in presence theoretical and practical lectures, but also the disparities between students able to access to IT technologies and those living in rural places where the technological facilities are not available [8].

The Indian survey highlighted how students also developed anxiety in facing exams and university, since the distance and the not attendance of the faculty made them disoriented in contacted strangers administrators [8]. Another similar study testing the experience of the students at the University of Jordan the majority of the students felt the missing of the practical sessions [9], agreeing on the promising possibilities of developing a hybrid e-learning educational system.

Therefore, all of the studies in literature reports how students consider fundamental the practical training for a full preparation to begin the dental profession.

### 4.2. The Educational Theories before the Pandemic

Traineeships are the core of dental education: the General Assembly of the Association for Dental Education in Europe (ADEE) has stated “dentists should be individuals who recognize problems in their professional practices and seek solutions by gaining skills through lifelong learning”. Unlike the sister discipline of Medicine, which can rely on the many “telemedicine” tools that are available to improve clinical education [10], Dentistry and Surgery need practical simulations [11]. Indeed, the delivery of professional skills in clinical education include a variety of theories that are applied according to the traits of the student characters, the educational tools, the laboratory provided and lastly, the outcome of the learning processes [12].

Training health profession students is a very particular aspect in education: the learners are adults and, therefore, they are responsible for their learning approaches. In the application of Knowles’ principles that guide the andragogy, the science behind the learning for adults, it is useful to understand how to teach and to help these types of learners [9]. According to the Knowles’ principles, the educator should establish a “safe” learning environment, where the students can feel free to express themselves, to be involved, to contribute to the setting of their learning goals and necessities and to develop self-criticism to promote continued skill improvement [13].

In Italian dental traineeships, two learning strategies are used: simulations on models and attendance at the university dental clinics or at affiliated dental centers. The first strategy relies on deliberate practice theory, which includes the moment of practicing of specific manual movements, with the possibility of making mistakes. The students can then learn by these mistakes, to acquire self-consciousness of their own limits and eventually to overcome them [14].

The second strategy instead relies on the theory of experiential learning. In particular, Kolb’s theory, which is based on six tenets, shows very clearly why the student must attend the dental clinics and how they are supposed to learn to be good practitioners [15]. According to Kolb’s theory, learning is a process, not a result and for each learning moment, the subject uses the previously gained knowledge to further build and extend this knowledge. Learning requires the resolution of a conflict between different ways of adaptation and the cognitive plane is only a part of this: learning includes the whole person actions and reactions [15]. It appears odd that learning is, therefore, the result of a synergistic interaction between the environment and the subject, a knowledge building due to the acquisition and transformation of the experience.

According to Kolb, learning is a social process during which people own a dynamic identity that is supported by experiences, educational moments and a safe environment [15]. Kolb described learning as a circular process that is divided into four stages [14]:

(1) Active and involving experience;
(2) Reflective observation, with elaboration of the experienced moments from many points of views;
(3) Abstract conceptualization, where the created concepts integrate the observations themselves;
(4) Active actualizations in new situations that are derived from the past experience.

Finally, the traineeships usually involve small groups of people who are supervised and led by a tutor or professor, with the obvious consequent social interactions. Bandura social learning theory [16] highlights the dynamics of the learning process, especially for self-confidence and the learning by observation of the failing of oneself or of others and by the success arising from the acquisition of new skills. Bandura identified three essential models:

(1) Live observation;
(2) Verbal instructions, with actions and behavioral actions;
(3) Symbolic model.

Therefore, the Bandura social learning model also includes the listening to podcasts, the learning from watching videos and so on [12]. Thus, the laboratory simulations train the practical skills and the clinical internships train the mind of the operator through the face-to-face interactions with the patients, colleagues and supervisors.

### 4.3. The Pandemic and Dental Education: Italy and Europe and Their Differences

Beyond changing the personal and social habits of most people, the COVID-19 pandemic opened a veritable Pandora’s box. In terms of the challenges for the provision of dental education, the criticisms raised due to the suspension of internships for undergraduate students seriously questioned the usual teaching and learning approaches and presented new challenges for both students and lecturers [17,18,19]. The online approach resulted in wider openings for the delivery of the theorical aspects of education, with a “call of duty” to the self-learning traits. However, the attempts to replace the internships with virtual case simulations (i.e., relying on the Bandura social learning theory) was not enough according to these perceptions of the students.

During the Italian dental curriculum, while the practical skills are gained through all of the course, this reaches a particular focus from the third year to the sixth year of the course. The organization of the sixth year, in particular, has been left to the autonomous organization of each institution and it is usually dedicated to the traineeships.

Interestingly, the relative level of the sixth-year significant feedback of “scarce” was lower than that from the other student year groups. In particular, this would appear to be due to the degree of maturity and experience of these sixth-year students.

The management of the consequences of the anti-COVID-19 measures on dental education have been faced in different ways across different countries. Even if the use of online lectures and e-learning tools has indeed been the general common strategy, the ways in which these have been used were various and have included live lectures or streaming of lectures, with videos, links to online applications for simulations and online assessments.

Mladenovic et al. (2020) reported positive experiences for the development and use of a mobile (telephone) application (App) for the dynamic study of dental traumatology. Indeed, in their study, the majority of the students reported very positive feedback for this App, as they appreciated the flexibility and the curiosity it induced [20]. Stoopler et al. (2020) reported their experience where a virtual hospital was used for practical training and also for student examinations for those about to graduate, with positive outcomes indicated [21]. Both of these recent studies have also highlighted how if used correctly, these digital technologies can offer wider series of tools that can also be applied to the teaching of the clinical practice.

Sajdłowski et al., reported how similarly in Poland the students appreciated the online lectures, but the traineeship and hands-on session constitute unique moments that cannot be replaced with e-learning [22].

The teaching strategy used at the “D”Annunzio” University of Chieti–Pescara was generally welcomed by the students for their main (theory) lectures. The other aspects that the students appreciated were the possibility to follow the lectures again (to better understand the concepts), the flexibility of their schedule (to manage their logistics) and the lack of associated problems (such as lack of light in the room, projector problems). A recent survey by Hung et al. (2020) also showed how dental students share concerns about their clinical education and their future, in terms of employment and recompense, which can add more stress to an already high-pressure profession [23].

Before the COVID-19 pandemic, in 2019, a study on the perceptions of students to e-learning approaches showed how online lectures and videos of clinical procedures were indeed felt to be helpful during their studies, but the idea of total replacement of practical sessions by these recordings was not welcomed [24].

### 4.4. Future Perspectives

For the future then, as after the COVID-19 pandemic and during the probable extended period of our co-existence with the virus, Dental Schools will need to capitalize on this experience and re-organize their e-teaching and e-learning approaches, as showed from the stakeholder mapping matrix. The peculiar moments of the traineeships thus need to be replaced by virtual reality systems, when social distancing is required for public health issues [18]. However, these advanced technologies are not available to every institution and, therefore, dental lecturers are required to be creative to provide the students with the best learning experiences they can and institutions must invest in the new technologies that can provide distance simulations, such as virtual and augmented realities [25]. From the stakeholder mapping matrix another category might be influenced in negative or in positive from the interruption of the traineeships: the insurances covering for dental malpractices. As stated Pinchi et al. an effective and valid practical training plays a key role to reduce the cases of the lawsuits in implant dentistry [26]. In addition, as from the student feedback here, improvements and investments are required to assure adequate connectivity. Dental educators should also be made aware of the learning strategies that can be adopted in case the training suspension cannot be completely removed to fully allow the practical apprenticeships [25].

Indeed, the strengths of these online lectures (i.e., flexibility, availability of recordings, better quality of lectures) should be used to improve the shifts and hours of clinical laboratories and clinical practice sessions. As small groups of people still need to be maintained to avoid crowding, the number of hours needed for practical teaching (i.e., clinical practices) has now increased. However, distance teaching during this period of co-existence with the virus now allows better scheduling for both students and lecturers, potentially with more time available to follow practical sessions in the clinic.

In addition, dental clinics need to be carefully monitored to be sure to offer safe places for carrying out dental treatment, for both patients and operators and for learning, for our students. The psychological well-being of the dental students and Dental Faculty staff needs to be carefully monitored too and the use of digital technologies as the workplace should be regulated.

## 5. Conclusions

The COVID-19 pandemic has presented dental educators with a difficult challenge, due to the practical nature of the dental profession. However, this tragic event, given the number of victims and the economic consequences, can provide us with hints on the ways in which we can improve the University teaching and learning approaches, using the technological innovations that are now more widely available.

## Figures and Tables

**Table 1:** Survey used for this study, translated into English for present understanding.

Question Number	Question
1	Consent acceptance
2	Gender
3	Age
4	Year of course
5	How would you describe the level of interest for the at-a-distance lectures through the platform we used?
6	How would you describe the interactivity levels during the lectures?
7	Have your expectations regarding the at-a-distance lecture contents been satisfied?
8	How would you describe the lecturers” efforts to guarantee the teaching even during the emergency period?
9	How would you describe the at-a-distance approaches to the aspects that require the development of practical skills?
10	How would you describe the cultural knowledge you have acquired during the COVID-19 emergency period?
11	How adequate would you describe your level of preparation compared to the lectures attended in the pre-COVID-19 period?
12	Points of strength of the at-a-distance lectures
13	Limitations of the at-a-distance lectures

**Table 2:** Kruskal–Wallis test results. The dependent variable was the year of course.

Question	Answer	*n*	Kruskal-Wallis Test		
			Chi-Square	DF	*p*-Value
Level of Interest	Fair	119	21.0816	4	0.0003
	Satisfying	96			
	Very Good	47			
	Excellent	30			
	Scarce	9			
Level of Interactivity	Fair	121	31.1323	4	<0.0001
	Satisfying	76			
	Very Good	33			
	Excellent	27			
	Scarce	44			
Correspondence to expectations	Fair	109	26.4313	4	<0.0001
	Satisfying	96			
	Very Good	33			
	Excellent	46			
	Scarce	17			
Lecturers’ efforts	Fair	63	6.3095	4	0.1772
	Satisfying	121			
	Very Good	59			
	Excellent	55			
	Scarce	3			
E-teaching approach to the practical matter	Fair	76	14.0712	4	0.0071
	Satisfying	61			
	Very Good	133			
	Excellent	17			
	Scarce	14			
Level of personal Knowledge	Fair	120	4.8898	4	0.2988
	Satisfying	98			
	Very Good	16			
	Excellent	47			
	Scarce	20			
Level of personal preparation	Fair	117	9.0823	4	0.0591
	Satisfying	46			
	Very Good	36			
	Excellent	84			
	Scarce	18			

**Table 3:** Most representative points raised by the students in the open-ended questions. Quotations are translated from Italian, for present understanding.

Strengths	Limitations
The e-learning and e-teaching in these difficult months have allowed us to continue our learning path. In addition, we have had more time available to dedicate to other activities (e.g., reading, fitness)	Lack of fundamental internships
Feasibility of timetables	Low quality internet connections
Possibility to follow the lectures again at different times during the day, as the presentations are available	Direct interactions with the lecturers are obviously low and for some courses it is fundamental to better understand some topics. Even though the lecturers tried their best to overcome this limitation, the normal approach is not the same. For example, it was not possible to use the microscopes and observe the histology slides.
Better concentration and the possibility to record the lectures	I am not happy about the internships. Indeed, the dental profession is extremely practical and after the COVID-19 pandemic, it will be urgent to reorganize to attend the clinic.

## Data Availability

Data are available upon reasonable request to the corresponding author.

## Supplementary Materials

The following are available online at https://www.mdpi.com/article/10.3390/healthcare9040454/s1, Figure S1: Stakeholder mapping of interest showing the categories interested by the new model of dental education during pandemic crisis.
