# Supplementary material for: Dental Education Challenges during the COVID-19 Pandemic Period in Italy: Undergraduate Student Feedback, Future Perspectives, and the Needs of Teaching Strategies for Professional Development
Source: Healthcare (Basel). 2021 Apr 12;9(4):454. doi: 10.3390/healthcare9040454 (PMC8069889; doi:10.3390/healthcare9040454)
Supplement: Supplementary file 1 [file healthcare-09-00454-s001.pdf]

Stakeholder mapping matrix – Dental education model during Pandemic crisis

Influences	Stakeholder category	Stakeholder group	Goals, motivations, and interests	Influence	Interest	Action	Win/win strategies
Governance	University	Senate and Chancellor's office	The Universities reputation and standing in terms of facing a pandemic crisis.	High	High	Key player	Sign off of key decisions and stages via existing channels. Motivating Academic, Administrator and Technical Staff in keep an high quality standard
		External Relations Office/Communications Office	Maintaining a positive public image for the university, its staff and students.	High	High	Key player	Partner in the development and delivery of the communication plan.
		Business and enterprise	Keep a good and health economic balance, Funding application for educational investment	High	High	Key player	Consultation on any projects that impact existing business partnerships; investment in the advanced facilities and qualified personnel to continue to deliver an appropriate dental education, both theorical and practical.
Providers	Suppliers	Catering<br>Maintenance<br>Cleaning	Ability to continue business as usual and potential impact on existing contracts. Improving the sanitizing procedures	Low	High	Show consideration	Show consideration via regular updates and provide clear channels for expressing concerns.
		IT Providers	Improving the quality of the platform for the delivering of e-learning educations and the increase of the advanced technologies such as the virtual reality for the practical simulations at home	High	High	Key player	Continue negotiation and improvement of annual subscription for the educational platforms
		Dental educational tool providers	Improving the hands-on activity and the realization of a traineeship at home in case of in presence suspensions	High	High	Key player	Contact e negotiation of contracts between university and the Dental educational tool providers to offer to each students the appropriate material to practice at home, with the supervision of the professor on the e-learning platform

Regulatory	Emergency services and utilities	Occupational health service	Assuring the health of the dental operators, including professors, dentists, dental assistants, tutors, residents and dental students in traineeship	High	High	Key player	Screening tests before the access to the structure, adequate furniture of personal protection equipment (PPE), program of vaccination, security courses on the prevention of infection in practice
Users/beneficiaries	Community	Patients	The standard of care delivered to the patients should not be lowered in quality	High	High	Show consideration	Considerations of the standard of qualities of the dental care delivered by future dentist, which are trained in a different way. Take the opportunity to develop a different and a better training program
		Insurances	Poorly trained dentists might be responsible for malpractices, and these types of increase should be avoided	Low	High	Show consideration	Show consideration for cooperation for sponsored hands-on course for advanced undergraduate and post-graduate students. Special insurance covering for the students graduated with an obliged different model of training
	Schools/Faculties/Departments	Academic staff	Improve the quality of the teaching approach, included the practical notions during pandemic crisis	High	High	Key player	Diversification of the teaching methods, regular updates on the facilities available for a better deliver of practical lectures. Strict schedule for the "in presence" practical activities
		Administrative staff	Support in the funding application for new and advanced facilities	Low	High	Show consideration	Providing to the teachers "tips&tricks" to simplify the application for the new equipment acquisition
		Technical staff	Support in the IT management	High	High	Key player	Increase in quantity and quality the IT technician staff to provide a better support to academic, administrative Staff and students
	Students	Students	Improve the adaptation to a new learning approach.	High	High	Key player	Students should not rely on the lecturer to stimulate the learning process, being responsible to review materials before class, including book chapters, videos, case studies.

© T Morphy 2013
www.stakeholdermap.com
